# Survey of Early Practices and Perceptions of Liver Machine Perfusion Among US Liver Transplant Surgeons

**DOI:** 10.1097/TXD.0000000000001841

**Published:** 2025-06-27

**Authors:** Michelle C. Nguyen, Xingjie Li, Chi Zhang, Stephanie Ohara, Mehrdad Motamed, Caroline C. Jadlowiec, Adyr A. Moss, Kunam S. Reddy, Amit K. Mathur

**Affiliations:** 1 Division of Transplant Surgery, Department of Surgery, Mayo Clinic Arizona, Phoenix, AZ.; 2 Department of Surgery, Washington University, St. Louis, MO.; 3 Department of Surgery, Mayo Clinic Arizona, Phoenix, AZ.; 4 Department of Quantitative Health Science Research, Mayo Clinic Arizona, Phoenix, AZ.

## Abstract

**Background.:**

Ex vivo machine perfusion (MP) has transformed organ preservation, offering significant benefits in liver transplantation (LT), particularly with high-risk donor grafts. However, adoption in the United States has been limited. We aimed to examine early adoption trends, surgeon perceptions, and barriers to implementing MP in the United States after Food and Drug Administration approval of MP platforms.

**Methods.:**

A 23-question electronic survey was distributed to members of the American Society of Transplant Surgeons between October and November 2022, capturing attitudes and practices related to MP adoption. Responses from 96 surgeons representing 77 LT centers across 11 Organ Procurement and Transplantation Network regions were analyzed.

**Results.:**

Forty-four respondents (48%) reported having an MP program at their institution. Adoption of MP was significantly more common in high-volume centers and those performing ≥20 donation after circulatory death (DCD) transplants annually (*P* < 0.001). MP utilization received strong support, with 88% endorsing its use for DCD liver allografts and 82% for donation after brain death allografts. Respondents cited MP’s ability to reduce ischemic cholangiopathy, enable graft repair, and facilitate viability assessment as key benefits. Normothermic MP was preferred for high-risk donor profiles, including DCD grafts, older donors, and steatotic livers, and was associated with an increased willingness to accept medically complex grafts compared with static cold storage. Barriers to MP utilization included program costs, personnel demands, and logistical complexities. Centers with higher proportions of privately insured patients were more likely to adopt MP. Despite these challenges, 84% of respondents expressed interest in future MP adoption.

**Conclusions.:**

MP enhances graft utilization and outcomes, particularly for complex and high-risk donor livers, but widespread US adoption requires addressing financial and logistical barriers. Future efforts should focus on refining cost-effectiveness analyses, collaboration with organ procurement organizations and device companies, and developing standardized training to optimize MP integration and maximize its clinical impact on LT.

## Graphical abstract

**Figure:**
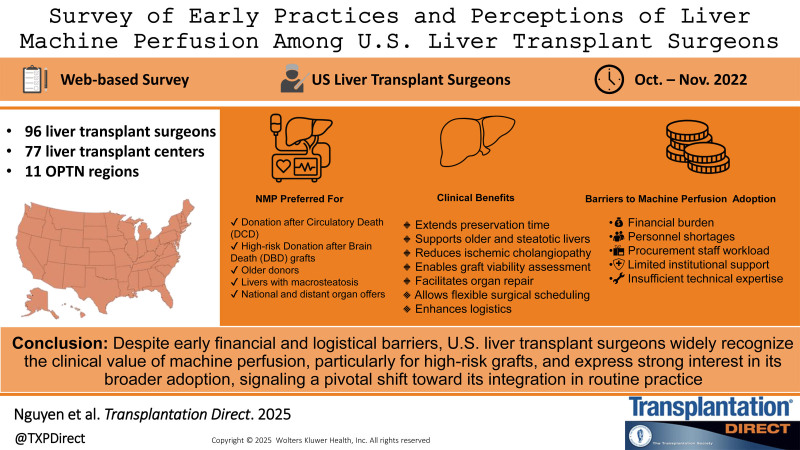

Ex vivo liver machine perfusion (MP), initially developed and trialed in Europe, has significantly evolved over the past decade, offering an innovative alternative to static cold storage (SCS) in organ preservation. The need for improved preservation methods is particularly evident with medically complex donor organs, high discard rates, and ischemia–reperfusion injury, which pose substantial challenges in liver transplantation (LT). Early studies by the Oxford Transplant group, demonstrated that normothermic MP (NMP) was safe and feasible, highlighting potential improvements in early liver dysfunction compared with SCS.^1^ Subsequent randomized clinical trials confirmed significant reductions in early allograft dysfunction (EAD), and liver discard rates, without compromising patient or graft survival.^2^ Similar findings from the US PROTECT trial highlighted that NMP reduced ischemic biliary complications and EAD rates, especially in donation after circulatory death (DCD) LT.^3^ The introduction of hypothermic MP (HMP) further supported MP’s benefits, demonstrating reduced biliary strictures in DCD transplants.^4^ Additionally, normothermic regional perfusion (NRP), an alternative approach that restores circulation before organ retrieval, has shown promise in mitigating ischemia–reperfusion injury and improving posttransplant outcomes, particularly in DCD donors.^5^

Despite this clinical evidence, the widespread implementation of MP faces significant barriers. Logistical complexities, including challenges in transportation, graft evaluation, and staff training, persist alongside financial concerns. The estimated cost for 1 disposable component of MP in the United States can range from $30 000 to $100 000 per LT, further contributing to the slow adoption.^6,7^ Furthermore, disparities in MP utilization between the United States and Europe, where MP was integrated more rapidly, underscore the need to address region-specific challenges.

International surveys have examined MP utilization and its perceived benefits among transplant programs worldwide. A study of 143 respondents from 23 countries reported that 54% used HMP and 46% used NMP. Respondents cited improved organ quality and logistical flexibility, with broad support for routine use of MP for extended criteria donor livers. HMP was preferred for increasing high-risk graft utilization and reducing ischemic cholangiopathy (IC).^8^ Similarly, another international survey of 64 transplant programs highlighted MP’s benefits in improving workflow efficiency, reducing staff burnout, and managing medically complex grafts.^9^ However, these studies often included international perspectives and devices not yet available in the United States.

This survey study captures a pivotal moment in the evolution of MP adoption in the United States. Since Food and Drug Administration (FDA) approval of 2 NMP devices in 2021, MP has gained broader acceptance, yet understanding early perceptions and practices remains essential for identifying persistent barriers and informing strategies wider implementation.^10,11^ By surveying LT centers across diverse Organ Procurement and Transplantation Network regions, this study provides foundational insights into US LT surgeons’ perspectives during MP’s early adoption phase.

## MATERIALS AND METHODS

### Survey Design

An electronic survey was conducted to assess the attitudes, perceptions, and practices of LT surgeons in the United States regarding ex vivo MP. Designed as a 23-question web-based survey hosted on Qualtrics, the questionnaire included questions about respondents’ roles, clinical programmatic details and practices (eg, transplant volume, median Model for End-Stage Liver Disease (MELD) score at transplant, involvement in clinical trials, and utilization of MP) (**Table S1, SDC,** https://links.lww.com/TXD/A776). The survey primarily utilized categorical multiple-choice questions, Likert scales for agreement, ordinal response options for decision-making, a numerical scale for barrier assessment, and open-ended fields for additional insights.

Clinical scenarios assessed respondents’ likelihood of accepting donor livers for an ideal recipient under varying donor risk factors. The scenarios were structured based on established clinical factors known to impact organ acceptance, including donor age (30, 55, or 65 y), graft type (donation after brain death [DBD] versus DCD), distance from the recipient center (within 250 versus beyond 500 nautical miles), and macrosteatosis percentage (<5% versus 20%–30%). These variables were selected based on prior literature, expert consensus, and discussions with experienced transplant surgeons to ensure clinical relevance. The donor characteristics were stratified to capture a range of risk profiles, from lower risk (eg, young DBD donors with minimal macrosteatosis) to higher risk (eg, older DCD donors and moderate macrosteatosis). The 250- and 500-nautical mile thresholds were chosen to reflect logistical challenges associated with organ transport and cold ischemia time (CIT).

To validate the clinical plausibility of the scenarios, they were reviewed by a panel of transplant surgeons and procurement specialists. The face validity of the scenarios was assessed by ensuring that they aligned with real-world transplant decision-making. Additionally, scenarios were iteratively refined based on feedback to optimize clarity and ensure that responses would capture meaningful variations in decision-making across different transplant centers. As detailed in **Table S2** (**SDC,** https://links.lww.com/TXD/A776), responses to each scenario were categorized into 3 decision outcomes: accept with SCS, accept with NMP, or decline. The analysis examined the willingness to accept or decline organs across various donor risk categories. **Table S2** (**SDC,** https://links.lww.com/TXD/A776) presents statistical comparisons of this willingness based on preservation methods and donor risk factors. The findings underscore how factors such as age, donor type, distance, and macrosteatosis influence decision-making. The survey also captured perceived barriers to MP adoption, with responses ranked on a scale from 0 (not a barrier) to 10 (extreme barrier).

The survey was distributed via email to 813 members of the American Society of Transplant Surgeons through the organization’s listserv between October 5 and November 8, 2022. Participation was voluntary and anonymous, with an estimated completion time of approximately 10 min. Because not all recipients were LT surgeons, the email invitation was explicitly directed at LT surgeons. This approach resulted in a response rate of 11.8% (96/813). Institutional review board approval (IRB22005249) was obtained, classifying the study as minimal risk research to human subjects.

### Statistical Analyses

Descriptive statistics were used to summarize respondent and program characteristics. Chi-square and Fisher exact tests were used to compare survey responses. For clinical scenario questions, pairwise comparisons were conducted to assess differences in respondents’ willingness to accept organs based on preservation method (SCS or NMP) or to decline offers, both within and between groups, across varying donor factors. To account for the correlation of the responses from the same respondent, generalized estimating equation approach was used so that the robust statistical inference was provided. All analyses were conducted using SAS version 9.4 (SAS Institute, Cary, NC), with statistical significance set at a two-sided α of 0.05.

## RESULTS

### Demographical Data

**FIGURE 1.**
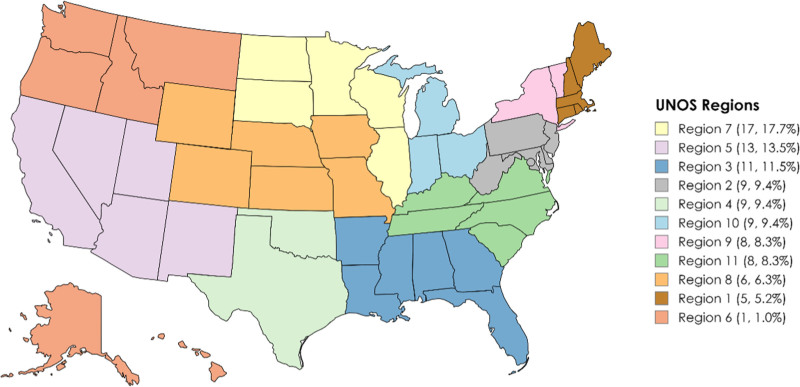
Map of Organ Procurement and Transplantation Network regions represented by the number of respondents.

**FIGURE 2.**
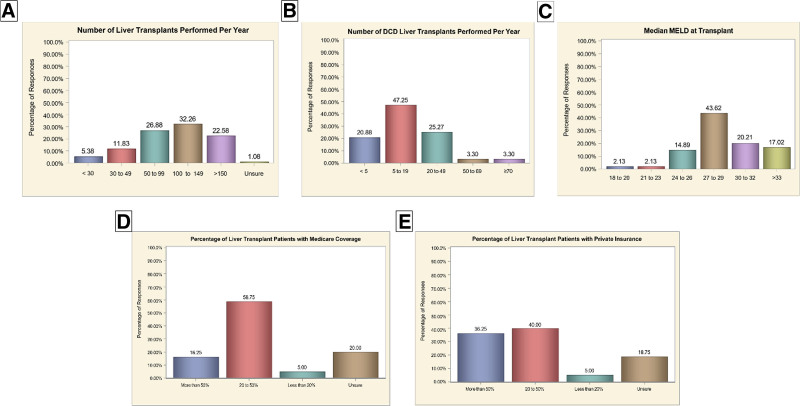
Institutional characteristics of participating transplant centers:(A) annual liver transplant volume; (B) DCD transplant volume; (C) median MELD at transplant; (D) proportion of patients with medicare coverage; (E) proportion of patients with private insurance coverage. DCD, donation after circulatory death; MELD, Model for End-Stage Liver Disease.

Ninety-six responses were received from LT surgeons, representing 77 centers across 11 Organ Procurement And Transplantation Network regions (Figure 1). Of these, 34 (36.2%) were surgical LT center directors. Fifty-one (54.9%) worked at high-volume centers performing >100 LTs annually, whereas 5 (5.4%) represented centers performing fewer than 30 LTs. Most respondents (47.3%) reported performing 5–19 DCD LTs annually, whereas 25.3% performed 20–49 DCD cases, and only 3.3% performed 50 or more. The median MELD at transplant (MMaT) scores varied, with 43.6% of respondents reporting MMaT of 27–29, 20.2% reporting scores of 30–32, and 17.0% reporting MMaT ≥33. Regarding healthcare coverage, 58.8% of respondents indicated Medicare coverage for 20%–50% of their patients, while 16.25% reported coverage exceeding 50%. For private insurance, 40.0% reported coverage for 20%–50% of patients, and 36.3% reported private insurance coverage for >50% (Figure 2).

### MP Utilization Practices

**FIGURE 3.**
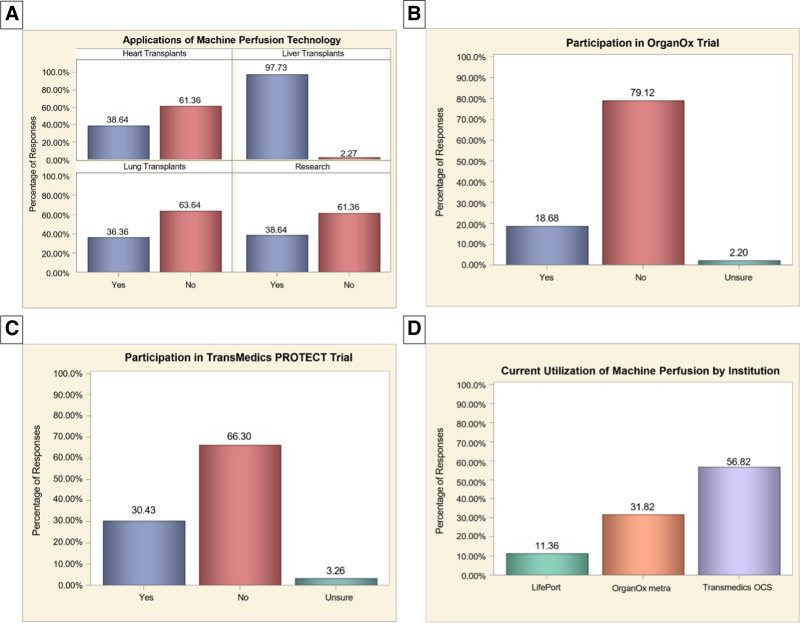
Clinical integration and research participation in machine perfusion across transplant programs: (A) current clinical applications of machine perfusion; (B and C) participation in machine perfusion–related clinical trials; (D) type of machine perfusion technology currently in use by institutions.

Forty-four respondents (48%) reported having a current active MP program at their institution. Among these, 97.7% applied MP for LT, 38.6% for heart transplants, 36.4% for lung transplants, and 38.6% for research purposes. Use of MP for research purposes, heart transplants, and lung transplants was significantly associated with having a liver MP program (*P* < 0.001). Seventeen (18.7%) respondents participated in the OrganOx clinical trial, whereas 28 (30.4%) participated in the TransMedics PROTECT clinical trial. The most used MP platform was TransMedics Organ Care System (OCS; 56.8%), followed by OrganOx metra (31.8%) and LifePort (11.4%) (Figure 3). Respondents from institutions with MP programs were significantly more likely to perform higher volumes of LTs annually (*P* < 0.01), with 41.9% performing 100–149 transplants and 32.6% performing ≥150 transplants compared with 26.1% and 10.9% of non-MP centers, respectively. Participation in the OrganOx trial was significantly associated with MP adoption (*P* = 0.01), with 31.8% of MP centers participating compared with 6.5% of non-MP centers. Most respondents (58.8%) reported that 20%–50% of their LT patients have Medicare coverage with no significant difference between centers with or without an MP program (*P* = 0.46). Similarly, 40% of centers reported 20%–50% of patients having private insurance coverage, although centers with active MP programs were more likely to report higher percentages of privately insured patients compared with those without an MP program (*P* = 0.001). Notably, centers without MP programs were more likely to report <20% private insurance coverage or to be unsure about their payor mix (Table 1).

**TABLE 1.** Association between presence of an ex situ machine perfusion program and programmatic characteristics

	Does your institution have an ex situ machine perfusion program?					
	Missing (N = 5)	Yes (N = 44)	No (N = 46)	Unsure (N = 1)	Total (N = 91)	*P*
How many liver transplants does your center perform per year? n (%)						<0.01^*a*^
Missing	2	1	0	0	1	
<30	0	0 (0.0)	5 (10.9)	0 (0.0)	5 (5.6)	
30–49	0	2 (4.7)	8 (17.4)	1 (100.0)	11 (12.2)	
50–99	1	9 (20.9)	15 (32.6)	0 (0.0)	24 (26.7)	
100–149	0	18 (41.9)	12 (26.1)	0 (0.0)	30 (33.3)	
150 or more	2	14 (32.6)	5 (10.9)	0 (0.0)	19 (21.1)	
Unsure	0	0 (0.0)	1 (2.2)	0 (0.0)	1 (1.1)	
Please select your center’s median MELD at transplant, n (%)						0.70^*a*^
18–20	0	2 (4.5)	0 (0.0)	0 (0.0)	2 (2.2)	
21–23	0	2 (4.5)	0 (0.0)	0 (0.0)	2 (2.2)	
24–26	1	8 (18.2)	5 (10.9)	0 (0.0)	13 (14.3)	
27–29	1	18 (40.9)	21 (45.7)	1 (100.0)	40 (44.0)	
30–32	0	8 (18.2)	11 (23.9)	0 (0.0)	19 (20.9)	
33 or more	1	6 (13.6)	9 (19.6)	0 (0.0)	15 (16.5)	
Was your institution involved in the TransMedics PROTECT trial? n (%)						0.27^*a*^
Yes	0	18 (40.9)	10 (21.7)	0 (0.0)	28 (30.8)	
No	1	24 (54.5)	35 (76.1)	1 (100.0)	60 (65.9)	
Unsure	0	2 (4.5)	1 (2.2)	0 (0.0)	3 (3.3)	
Is your institution currently involved in the OrganOx trial? n (%)						0.01^*a*^
Yes	0	14 (31.8)	3 (6.5)	0 (0.0)	17 (18.7)	
No	0	28 (63.6)	43 (93.5)	1 (100.0)	72 (79.1)	
Unsure	0	2 (4.5)	0 (0.0)	0 (0.0)	2 (2.2)	
Approximately what percentage of your liver transplant patients have Medicare coverage? n (%)						0.46^*a*^
Missing	5	5	6	0	11	
<20%	0	2 (5.1)	2 (5.0)	0 (0.0)	4 (5.0)	
20%–50%	0	22 (56.4)	25 (62.5)	0 (0.0)	47 (58.8)	
>50%	0	6 (15.4)	6 (15.0)	1 (100.0)	13 (16.3)	
Unsure	0	9 (23.1)	7 (17.5)	0 (0.0)	16 (20.0)	
Approximately what percentage of your liver transplant patients have private insurance? n (%)						<0.001^*a*^
Missing	5	5	6	0	11	
Less than 20%	0	0 (0.0%)	3 (7.5%)	1 (100.0%)	4 (5.0%)	
20 to 50%	0	16 (41.0%)	16 (40.0%)	0 (0.0%)	32 (40.0%)	
More than 50%	0	14 (35.9%)	15 (37.5%)	0 (0.0%)	29 (36.3%)	
Unsure	0	9 (23.1%)	6 (15.0%)	0 (0.0%)	15 (18.8%)	

^*a*^Chi-square *P* value.

MELD, Model for End-Stage Liver Disease.

### Associations With DCD Transplant Volume and NRP Utilization

Institutions performing more DCD transplants were significantly more likely to have MP programs (*P* < 0.01). MP adoption increased from 21.1% in centers performing <5 DCD transplants annually to 73.9% for those performing 20–49 transplants and 100% for those performing ≥50. The frequency of NRP use was strongly associated with DCD volumes (*P* < 0.001), with NRP adoption exceeding 25% in 66.7% of respondents performing ≥70 DCD transplants annually (Table 2). Most respondents (72%) agreed that there are benefits of MP for NRP-recovered DCD livers.

**TABLE 2.** Association between DCD liver transplant volume and perspectives on machine perfusion and normothermic regional perfusion utilization

	How many DCD liver transplants does your center perform per year?							
	Missing (N = 5)	Less than 5 (N = 19)	5–19 (N = 43)	20–49 (N = 23)	50–69 (N = 3)	≥70 (N = 3)	Total (N = 91)	*P*
Does your institution have an MP program? n (%)								<0.01^*a*^
Yes	0	4 (21.1)	17 (39.5)	17 (73.9)	3 (100.0)	3 (100.0)	44 (48.4)	
No	0	15 (78.9)	26 (60.5)	5 (21.7)	0 (0.0)	0 (0.0)	46 (50.5)	
Unsure	0	0 (0.0)	0 (0.0)	1 (4.3)	0 (0.0)	0 (0.0)	1 (1.1)	
How often do you use NRP for DCD liver procurements? n (%)								<0.001
Never	0	16 (84.2)	14 (32.6)	5 (21.7)	0 (0.0)	0 (0.0)	35 (38.5)	
<5%	0	2 (10.5)	15 (34.9)	11 (47.8)	3 (100.0)	0 (0.0)	31 (34.1)	
5%–10%	0	1 (5.3)	7 (16.3)	2 (8.7)	0 (0.0)	1 (33.3)	11 (12.1)	
11%–25%	0	0 (0.0)	3 (7.0)	3 (13.0)	0 (0.0)	0 (0.0)	6 (6.6)	
>25%	0	0 (0.0)	4 (9.3)	2 (8.7)	0 (0.0)	2 (66.7)	8 (8.8)	
How much do you agree or disagree with the following statement: there are benefits of utilizing ex vivo machine perfusion for DCD liver transplants, n (%)								0.27^*a*^
Missing	5	0	2	3	0	0	5	
Strongly agree	0	5 (26.3)	21 (51.2)	13 (65.0)	2 (66.7)	3 (100.0)	44 (51.2)	
Agree	0	13 (68.4)	19 (46.3)	7 (35.0)	1 (33.3)	0 (0.0)	40 (46.5)	
Disagree	0	1 (5.3)	1 (2.4)	0 (0.0)	0 (0.0)	0 (0.0)	2 (2.3)	
How much do you agree or disagree with the following statement: there are benefits of utilizing ex vivo machine perfusion for DCD livers recovered with NRP, n (%)								0.73^*a*^
Missing	5	0	2	2	0	0	4	
Strongly agree	0	2 (10.5)	11 (26.8)	2 (9.5)	0 (0.0)	1 (33.3)	16 (18.4)	
Agree	0	11 (57.9)	21 (51.2)	12 (57.1)	1 (33.3)	2 (66.7)	47 (54.0)	
Disagree	0	5 (26.3)	8 (19.5)	6 (28.6)	2 (66.7)	0 (0.0)	21 (24.1)	
Strongly disagree	0	1 (5.3)	1 (2.4)	1 (4.8)	0 (0.0)	0 (0.0)	3 (3.4)	

^*a*^Chi-square *P* value.

DCD, donation after circulatory death; NRP, normothermic regional perfusion.

### Perceived Benefits of MP

**FIGURE 4.**
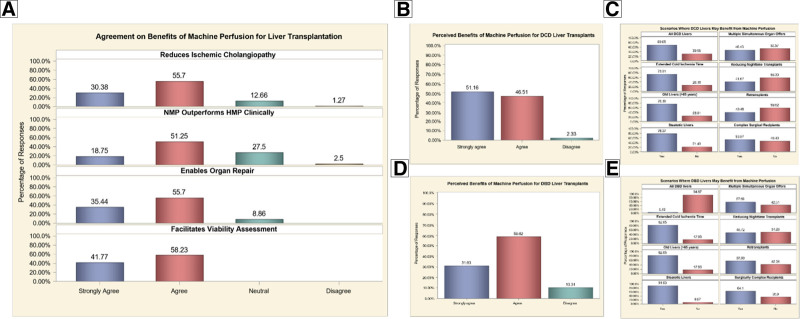
Perceived benefits and utilization of machine perfusion in liver transplantation. Agreement among respondents on the benefits of machine perfusion (A) and its utilization in DCD (B and C) and DBD (D and E) liver transplantation. DBD, donation after brain death; DCD, donation after circulatory death.

Respondents reported strong agreement on the benefits of MP for LT. Most respondents agreed that MP reduces IC (86.1%), enables organ repair (91.1%), facilitates viability assessment (100%) and that NMP outperforms HMP (70%) (Figure 4A). Respondents reported strong agreement on the benefits of MP for both DCD and DBD LTs. For DCD livers, 97.7% of respondents strongly agreed or agreed that MP provides benefits, whereas only 2.3% disagreed (Figure 4B). Similarly, for DBD livers, 89.6% agreed, and 10.3% disagreed (Figure 4D). For DCD livers, high agreement was observed for utilization of MP for all DCD transplants (69.1%) including scenarios such as steatotic livers (78.6%), livers from donors >65 y of age (76.2%), and livers with extended CIT (73.8%) (Figure 4C). In contrast, only 5% of respondents agreed that MP should be used for all DBD livers. For DBD livers, MP was seen as highly beneficial for steatotic livers (91.0%), livers from older donors (82.1%), and livers with extended CIT (82.1%) (Figure 4E).

### Willingness to Accept Organ Offers Across Clinical Scenarios and Preservation Strategies

**FIGURE 5.**
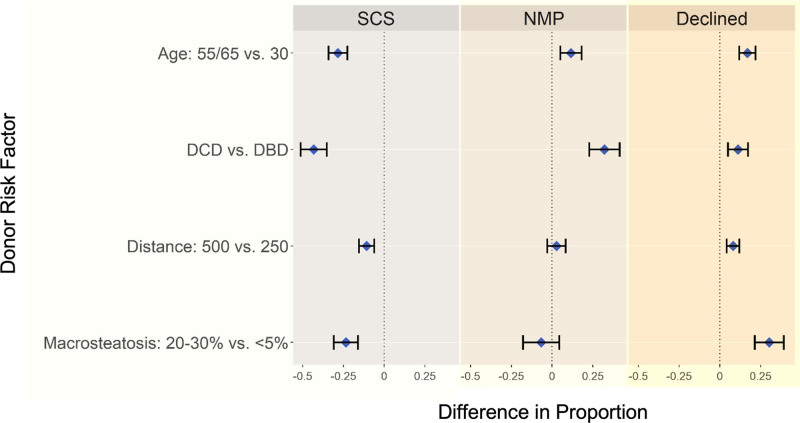
Variability in willingness to accept organs based on preservation strategy (SCS, NMP, declined) across different donor risk factors. NMP, normothermic machine perfusion; SCS, static cold storage.

The survey assessed willingness to accept organ offers across varying clinical scenarios, analyzing differences in respondent preferences for SCS, NMP, or declining the offer based on donor age, graft type, distance from the recipient center, and macrosteatosis level (**Table S2, SDC,** https://links.lww.com/TXD/A776). The willingness to accept organs from older grafts (55 or 65 years) was significantly lower with SCS compared with younger grafts (30 y), with a difference in proportion of −0.284 (95% confidence interval [CI]: −0.341 to −0.227; *P* < 0.001). In contrast, the willingness to accept organs from older grafts was higher with NMP (0.115; 95% CI: 0.050-0.181; *P* < 0.001). Decline rates were also higher for older donors (0.169; 95% CI: 0.119-0.219; *P* < 0.001). Graft type significantly influenced willingness to accept organ offers, with DCD grafts having been less likely to be accepted under SCS compared with DBD grafts (−0.432; 95% CI: −0.512 to −0.352; *P* < 0.001). However, the use of NMP significantly increased willingness to accept DCD grafts (0.321; 95% CI: 0.228-0.414; *P* < 0.001). The likelihood of declining DCD grafts was also significantly higher compared with DBD grafts (0.111; 95% CI: 0.049-0.173; *P* < 0.001). Grafts procured from distances >500 nautical miles were less likely to be accepted under SCS (−0.108; 95% CI: −0.154 to −0.061; *P* < 0.001). However, NMP did not significantly influence the likelihood of accepting grafts based on distance (0.027; 95% CI: −0.029 to 0.082; *P* = 0.350). Grafts with higher macrosteatosis (20%–30%) were less likely to be accepted with SCS compared with those with <5% macrosteatosis (−0.235; 95% CI: −0.310 to −0.160; *P* < 0.001). In contrast, for grafts preserved with NMP, macrosteatosis did not significantly affect willingness to accept (−0.067; 95% CI: −0.179 to 0.045; *P* = 0.240). Additionally, the likelihood of declining grafts increased significantly with higher macrosteatosis (0.303; 95% CI: 0.214 to 0.392; *P* < 0.001) (Figure 5, **Table S3a, SDC,** https://links.lww.com/TXD/A776).

**Table S3b** (**SDC,** https://links.lww.com/TXD/A776) highlights the statistical comparisons between preservation strategies. Significant differences were observed between SCS and NMP for donor age (*P* < 0.001) graft type (*P* < 0.001), donor distance (*P* = 0.010), and macrosteatosis level (*P* = 0.046), indicating that respondents favored NMP for higher-risk grafts. Comparisons between SCS and declined offers showed significant differences across all donor risk factors, indicating that respondents were more inclined to decline higher-risk grafts rather than accept them for preservation with SCS. Comparisons between NMP and declined offers showed significant differences for graft type (*P* = 0.004) and macrosteatosis level (*P* = 0.015), indicating that respondents were more inclined to accept higher-risk grafts to NMP rather than decline the offer.

### Barriers and Future Adoption

Financial burden was identified as the most significant barrier to MP adoption (median score: 8.0; interquartile range [IQR]: 4.0–10.0), followed by personnel shortages (median: 5.5; IQR: 0.0–7.5), procurement staff workload (median: 5.0; IQR: 2.0–7.0), limited institutional support (median: 5; IQR: 1.0–7.0), and insufficient technical expertise (median: 2.0; IQR: 0.0–5.5). Among respondents without MP programs, 84% indicated they were likely or very likely to adopt MP in the future.

## DISCUSSION

This study provides an overview of early adoption trends and perceptions of ex vivo MP among US LT surgeons. The findings strongly highlight surgeon agreement on the potential for MP to optimize organ preservation, particularly for high-risk DBD and DCD grafts. High-volume centers, those with more experience in DCD transplants, and participants in the OrganOx clinical trial were more likely to have active MP programs. Financial barriers and logistical challenges were identified as key constraints to widespread MP adoption. Despite these obstacles, 84% of respondents expressed interest in future MP adoption, emphasizing the need for targeted solutions to support broader implementation.

The adoption of liver MP in US clinical practice was initially hindered by delayed FDA approval and logistical challenges, including the geographic distribution of donor hospitals.^12^ Approval of the Transmedics OCS and OrganOx *metra* in 2021 marked a turning point, driving rapid clinical uptake.^12^ Within the first year, these data indicate respondents recognized the clinical potential of MP to reduce IC, enable organ repair, and facilitate viability assessment. Surgeons agreed that MP was particularly beneficial for extended criteria DBD and DCD grafts, especially grafts from older donors, with steatosis, and procured with over longer distances. Response validation through clinical scenarios confirmed these preferences, with respondents consistently favoring MP over SCS for higher-risk donor profiles. Notably, in donor DCD and macrosteatosis scenarios, respondents were more likely to accept these offers on NMP than decline the liver, indicating that they perceived NMP as an option to salvage some higher-risk grafts. The preference for MP in complex cases underscores its emerging importance in expanding the donor pool and enhancing the viability of medically complex grafts, as corroborated by subsequent clinical reports.^13-15^ At the time of our survey, 69% of respondents indicated that all DCD livers would benefit from MP.

Our findings are consistent with those of a similar international survey of 143 respondents from 23 countries, including 15 from the United States, that explored trends and barriers to the implementation of dynamic liver perfusion technologies. Notably, the survey was conducted between January and April 2021, before FDA approval of any MP devices in the United States. Among the respondents, 54% reported using HMP, and 46% reported using NMP, with the primary anticipated benefits being improved organ quality and enhanced logistical flexibility.^7^ The majority indicated they would consider routine use of MP for all extended criteria donor livers, with HMP regarded as the preferred platform to increase utilization of higher-risk organs and reduce the risk of IC.

HMP is more widely adopted in European countries than in the United States, largely because of earlier regulatory approvals and the availability of clinical data supporting its efficacy in reducing ischemic complications. European regulatory bodies approved devices, such as the Liver Assist and VitaSmart systems much earlier, allowing transplant centers in Europe to integrate HMP into routine practice. In addition to its clinical benefits, HMP is perceived as logistically simpler and less resource-intensive than NMP because it does not require continuous blood-based perfusate. In contrast, HMP devices have not been approved by the FDA at this point, limiting their adoption. Notably, in our survey, 11% of respondents with active MP programs reported using the LifePort system, likely in studies. The LifePort device is currently undergoing clinical trials in the United States to assess its safety and efficacy in LT. In contrast, the VitaSmart hypothermic oxygenated perfusion (HOPE) trial has been completed, demonstrating significant benefits including a reduction in EAD, shorter hospital stays, fewer major complications, and lower rates of IC and graft failure in DCD livers, with no device-related adverse events. These results are anticipated to support FDA approval and may broaden the availability of hypothermic perfusion platforms in the United States. Regulatory approval of HMP devices could help address key logistical and economic barriers currently limiting broader adoption of MP, assuming clinical outcomes are comparable to those achieved with normothermic preservation.

Our survey revealed that higher-volume transplant centers, particularly those performing >100 LT annually or >20 DCD LT per year, were more likely to adopt MP programs. This finding aligns with international data suggesting that high-volume centers have both the expertise and institutional support necessary to overcome the challenges associated with MP implementation.^8,9^ In the United States, DCD LTs utilization is heterogenous and clustered in few high-utilization centers, and these centers have developed risk appetite and expertise with riskier livers. These centers may also be experienced in understanding the burden of associated complications, prompting the need for and development of a MP program.^16^ Similarly, the results from this survey indicated that NRP utilization was associated with higher DCD volume. NRP has been shown to significantly reduce rates of IC, EAD, and biliary complications with comparable patient and graft survival to DBD transplants.^5,17-20^ This analysis reflects surgical enthusiasm for this technique, with most respondents (72%) also recognizing the potential benefits of MP for DCD organs recovered via NRP. In a subsequent study by Croome et al, outcomes after sequential NRP with NMP for DCD LT was compared with NRP alone and SCS. Among 83 DCD transplants, 0% of NRP and NRP + NMP cases had IC compared with 16.8% in the SCS group. Sequential NRP + NMP was used for medically complex grafts, long-distance recoveries, or complex recipients and demonstrated lower rates of EAD, reduced transfusion needs, and improved graft survival. The study highlights the complementary use of NRP and NMP to enhance organ viability and optimize outcomes in challenging cases. Given its promising impact on improving organ utilization and posttransplant outcomes, broader implementation of NRP across transplant centers could further expand the donor pool and reduce waitlist mortality.

More than one-third of respondents emphasized the logistical advantages of MP in LT, particularly for managing multiple simultaneous organ offers, enabling daytime transplants, and optimizing care for retransplant and complex surgical recipients across both DCD and DBD donors. These logistical advantages have been reported in the literature, with studies demonstrating that NMP can extend preservation times and shift transplant procedures from overnight emergencies to planned daytime surgeries.^21^ In another survey of 64 transplant programs, respondents recognized improved workflow efficiency, reduced staff burnout, and enhanced ability to manage medically complex grafts as key logistical benefits.^9^ This was confirmed in a large clinical study of NMP cases, demonstrating the benefits of NMP even for DBD grafts to facilitate concurrent organ offers, sequential transplants, and optimize logistics in high-risk or complex cases.^15^

Despite its clinical benefits, financial and logistical barriers remain significant obstacles to widespread adoption of MP. In the present survey, respondents identified program cost as the primary challenge in establishing and sustaining an MP program, followed by personnel demands and increased staff workload. These concerns are consistent with previous survey findings, which highlight upfront costs and operational complexity as key deterrents to implementation.^7,9,22^ However, recent real-world analyses suggest that the logistical efficiencies and complication reductions associated with MP may offset its higher acquisition costs. By reducing ischemia–reperfusion injury and biliary complications, NMP is projected to lower annual intervention costs by as much as $39 710.^3,23^ Over the long term, NMP could become the more economical approach to managing end-stage liver disease based on Markov modeling studies. One model showed that NMP yields greater quality-adjusted life years over 5 y (3.48 versus 3.17) compared with SCS alone, with lower mean costs ($456 455 versus $519 222), and was cost-effective in 63% of probabilistic sensitivity analysis iterations at a willingness-to-pay threshold of $40 941.^24^ Another demonstrated that NMP could improve utilization of discarded livers, increasing the annual yield of transplantable grafts by 5.8% and achieving an incremental cost-effectiveness ratio of $33 575 per additional quality-adjusted life year over a 10-year horizon.^25^

Real-world studies further support NMP’s cost-effectiveness, showing that it can offset its higher acquisition costs through logistical efficiencies. For instance, NMP has been associated with reduced nonutilization rates and therefore lower transportation costs for DCD livers by reducing “dry runs.”^26^ Webb et al^24^ reported that incorporating the OrganOx metra device into clinical practice shortened the waitlist and improved posttransplant survival. Similarly, Raigani et al^27^ found that the median cost to identify a viable graft using NMP was comparable to the monthly care costs for waitlisted patients with high MELD scores. A multicenter study further corroborated these findings, showing that implementing a routine NMP program reduced waitlist time and mortality without compromising short term survival, despite increased use of higher-risk allografts.^28^ Despite higher organ acquisition and preservation costs, studies indicate that 90-d healthcare expenses with NMP are comparable to SCS, primarily because of reduced posttransplant complications and improved outcomes.^29^ These findings suggest that NMP could achieve cost-effectiveness, particularly in a back-to-base modes. Nevertheless, further research is needed to evaluate device-to-donor approaches and optimize strategies for widespread adoption.

However, economic advantages may not be immediately apparent during early implementation. Although NMP reduces downstream costs, these savings may take time to materialize and transplant program/hospital finance relationships are complex. Reimbursement for additional organ acquisition costs further complicates adoption in the United States, as there is a diverse third-party payor environment.^12^ Center payer mix seemed to affect NMP adoption, with higher density of private insurance being associated with higher likelihood to adopt NMP (*P* < 0.001).

This study has several limitations. First, the survey targeted members of the American Society of Transplant Surgeons, which may have introduced selection bias. Respondents likely represent a subset of surgeons and transplant centers with a greater interest or involvement in advanced technologies, potentially skewing the results toward high-volume centers or those with more experience in using medically complex grafts. Consequently, the findings may not fully reflect the perspectives or practices of smaller or less-resourced centers. Second, the survey captures surgeon perceptions and hypothetical practices rather than actual clinical data, which could result in discrepancies between reported preferences and real-world implementation. Response options for organ acceptance in clinical scenarios were mutually exclusive and did not include combinations such as NRP + SCS, NRP + NMP, or HOPE, as HOPE was not commercially available in the United States at the time of the survey, and NRP was not widely adopted. The survey aimed to capture primary decision-making patterns rather than complex, multipronged strategies, ensuring standardization and comparability across donor risk factors. Although some centers may consider multiple preservation approaches, the structured response format allowed for clearer statistical analysis of clinical preferences within the existing technological landscape. Additionally, the study does not account for regional variations in organ procurement practices, donor characteristics, or financial resources, which can significantly influence MP adoption and utilization. For example, geographic disparities in access to donor hospitals and specialized transport systems could affect the feasibility of integrating MP in certain areas. Finally, because the survey provides a snapshot from the late 2022, it does not reflect subsequent developments in clinical practice, technology adoption, and organ utilization that have since reshaped the landscape of liver MP.

Since the survey was conducted in the late 2022, MP practices in LT have evolved considerably, with national data showing expanded adoption and access. The proportion of livers recovered from DCD donors increased from approximately 13% in early 2022 to 35% by early 2025. MP use in DCD livers rose from 2% to 52% during this period, while utilization in DBD livers increased from 0% to 18%. Concurrently, the nonutilization rate for DCD livers declined from 78% to 55%.^30^ Implementation of the national OCS program, with dedicated TransMedics-employed procurement surgeons, has further expanded access to MP by enabling more centers, including those without in-house MP infrastructure, to incorporate this technology during organ recovery and transport. In parallel, reimbursement pathways for NMP have gradually improved, with many centers now incorporating MP costs into organ acquisition charges or securing coverage through local Medicare administrative contractors and select commercial payers, although variability remains. In addition to center-level adoption, organ procurement organizations have increasingly contributed to MP expansion by supporting logistics, facilitating back-to-base perfusion strategies, and incorporating MP into recovery workflows.

In conclusion, MP has become a critical tool in LT, demonstrating its potential to expand the donor pool and improve outcomes, particularly for medically complex grafts. However, barriers remain, including cost, logistics, and inconsistent reimbursement, which must be addressed to support broader clinical integration. Future efforts should focus on refining cost-effectiveness analyses, expanding regulatory approval of hypothermic platforms, and developing standardized training programs to address operational challenges. Although head-to-head comparisons of NMP and HMP platforms would help optimize clinical use, these are unlikely to be industry-sponsored, underscoring the need for independently funded research. Coordinated efforts across stakeholders will be essential to ensure equitable adoption and to realize the full clinical and economic potential of MP in LT.

## Supplementary Material


