# Supplementary material for: Survey of Early Practices and Perceptions of Liver Machine Perfusion Among US Liver Transplant Surgeons
Source: Transplant Direct. 2025 Jun 27;11(7):e1841. doi: 10.1097/TXD.0000000000001841 (PMC12208642; doi:10.1097/TXD.0000000000001841)
Supplement: Supplementary file 1 [file txd-11-e1841-s001.pdf]

Table S1: Survey Instrument on Liver Machine Perfusion Practices and Perceptions Among Transplant Surgeons

Q1 **Please select your Organ Procurement and Transplantation Network (OPTN) region:**

○ Region 1 (1)

○ Region 2 (2)

○ Region 3 (3)

○ Region 4 (4)

○ Region 5 (5)

○ Region 6 (6)

○ Region 7 (7)

○ Region 8 (8)

○ Region 9 (9)

○ Region 10 (10)

○ Region 11 (11)

Q2 **What role do you serve in your Liver Transplant Program?**

Display This Choice:

If Q1 = 1

And Q1 != 1

○ Medical Director (2)

○ Liver Transplant Surgeon (4)

○ Surgical Director of Liver Transplant (1)

○ Other, please specify: (3)

____

Page Break

Q3 **How many liver transplants does your center perform per year?**

- ◯ Less than 30 (1)
- ◯ 30 to 49 (2)
- ◯ 50 to 99 (3)
- ◯ 100 to 149 (4)
- ◯ 150 or more (5)
- ◯ Unsure (6)

Q4 **Please select your center's median MELD at transplant.**

- ◯ 18 to 20 (1)
- ◯ 21 to 23 (2)
- ◯ 24 to 26 (3)
- ◯ 27 to 29 (4)
- ◯ 30 to 32 (5)
- ◯ 33 or more (6)

Page Break

Q5 **Was your institution involved in the Transmedics PROTECT trial?**

- ◯ Yes (1)
- ◯ No (2)
- ◯ Unsure (3)

Q6 **Is your institution currently involved in the OrganOx trial?**

- ◯ Yes (1)
- ◯ No (2)
- ◯ Unsure (3)

Q7 **Does your institution have an ex situ machine perfusion program?**

- ◯ Yes (1)
- ◯ No (2)
- ◯ Unsure (3)

Display This Question:

If Q7 = 1

Q8 **For which of the following is it used?** (Select all that apply.)

- ☐ Research purposes (1)
- ☐ Liver transplants (2)
- ☐ Heart transplants (3)
- ☐ Lung transplants (4)

Display This Question:

If Q7 = 1

Q9 **What ex vivo machine perfusion technology is currently utilized at your institution?**

- ○ Transmedics OCS (1)
- ○ OrganOx metra (2)
- ○ LifePort (3)
- ○ Other, please specify: (4) ____
- ○ None (5)

Page Break

Q10 **How many donation after circulatory death (DCD) liver transplants does your center perform per year?**

- ○ Less than 5 (1)
- ○ 5 to 19 (2)
- ○ 20 to 49 (3)
- ○ 50 to 69 (4)
- ○ 70 or more (5)
- ○ Unsure (6)

Q11 **How often do you use normothermic regional perfusion (NRP) for DCD liver procurements?**

- ○ Never (1)
- ○ Less than 5% (2)
- ○ 5 to 10% (3)
- ○ 11 to 25% (4)
- ○ More than 25% (5)

Page Break

Q12 **How much do you agree or disagree with the following statement: There are benefits of utilizing ex vivo machine perfusion for DCD liver transplant.**

- Strongly agree (1)
- Agree (2)
- Disagree (3)
- Strongly disagree (4)

Display This Question:

If Q12 = 1

Or Q12 = 2

Q13 **Which of the following DCD livers would benefit from ex vivo machine perfusion?** (Select all that apply.)

- Steatotic livers (1)
- Old livers (2)
- Long cold ischemia time (3)
- Retransplants (4)
- Surgically complex recipients (5)
- Nighttime transplants (6)
- Entertaining multiple simultaneous offers (7)
- All DCD livers (8)
- Other, please specify: (9) ____

Q14 **How much do you agree or disagree with the following statement: There are benefits of utilizing ex vivo machine perfusion for DBD liver transplant.**

- Strongly agree (1)
- Agree (2)
- Disagree (3)
- Strongly disagree (4)

Display This Question:

If Q14 = 1

Or Q14 = 2

Q15 **Which of the following DBD livers would benefit from ex vivo machine perfusion?** (Select all that apply.)

- ☐ Steatotic livers (1)
- ☐ Old livers (2)
- ☐ Long cold ischemia time (3)
- ☐ Retransplants (4)
- ☐ Surgically complex recipients (5)
- ☐ Nighttime transplants (6)
- ☐ Entertaining multiple simultaneous offers (7)
- ☐ All DBD livers (8)
- ☐ Other, please specify: (9) ____

Q16 **How much do you agree or disagree with the following statement: There are benefits of utilizing ex vivo machine perfusion for DCD livers recovered with NRP.**

- ○ Strongly agree (1)
- ○ Agree (2)
- ○ Disagree (3)
- ○ Strongly disagree (4)

Page Break

Q17 **The following clinical case scenarios describe liver organ offers. Please indicate your likelihood/interest of accepting these organ offers for an ideal recipient.**

	Accept with standard cold storage (1)	Accept with machine perfusion (2)	Decline offer (3)
**30 year** old **DBD** donor **WITHIN** 250 nautical mile acuity circle. Labs and CT imaging are normal. Procurement biopsy with **20 to 30% macrosteatosis**. (Q17_1)	○	○	○
**30 year** old **DBD** donor **BEYOND** 500 nautical mile acuity circle. Labs and CT imaging are normal. Procurement biopsy with **20 to 30% macrosteatosis**. (Q17_2)	○	○	○
**65 year** old **DBD** donor **WITHIN** 250 nautical mile acuity circle. Labs and CT imaging are normal. Procurement biopsy with **20 to 30% macrosteatosis**. (Q17_3)	○	○	○
**65 year** old **DBD** donor **BEYOND** 500 nautical mile acuity circle. Labs and CT imaging are normal. Procurement biopsy with **20 to 30% macrosteatosis**. (Q17_4)	○	○	○

**30 year** old **DCD** donor **WITHIN** 250 nautical mile acuity circle. Normal labs and imaging are normal. WIT 15 minutes. Procurement biopsy with **20 to 30% macrosteatosis**. (Q17_5)	○	○	○
**30 year** old **DCD** donor **BEYOND** 500 nautical mile acuity circle. Normal labs and imaging are normal. WIT 15 minutes. Procurement biopsy with **20 to 30% macrosteatosis**. (Q17_6)	○	○	○
**55 year** old **DCD** donor **WITHIN** 250 nautical mile acuity circle. Normal labs and imaging are normal. WIT 15 minutes. Procurement biopsy with **less than 5% macrosteatosis**. (Q17_7)	○	○	○
**55 year** old **DCD** donor **WITHIN** 250 nautical mile acuity circle. Normal labs and imaging are normal. WIT 15 minutes. Procurement biopsy with **20 to 30% macrosteatosis**. (Q17_8)	○	○	○

**55 year** old **DCD** donor **BEYOND** 500 nautical mile acuity circle. WIT 15 minutes. Procurement biopsy with **less than 5% macrosteatosis**. (Q17_9)	○	○	○
**55 year** old **DCD** donor **BEYOND** 500 nautical mile acuity circle. WIT 15 minutes. Procurement biopsy with **20 to 30% macrosteatosis**. (Q17_10)	○	○	○
**65 year** old **DCD** donor **WITHIN** 250 nautical mile acuity circle. **WIT 30 minutes**. Procurement biopsy with **20 to 30% macrosteatosis**. (Q17_11)	○	○	○
**65 year** old **DCD** donor **BEYOND** 500 nautical mile acuity circle. **WIT 30 minutes**. Procurement biopsy with **20 to 30% macrosteatosis**. (Q17_12)	○	○	○

Page Break

Q18 **How much do you agree or disagree with the following statements:**

	Strongly agree (1)	Agree (2)	Disagree (3)	Strongly disagree (4)
Normothermic machine perfusion is clinically superior to hypothermic machine perfusion. (Q18_1)	○	○	○	○
Ex vivo liver machine perfusion has the potential to decrease rates of ischemic cholangiopathy. (Q18_2)	○	○	○	○
Ex vivo liver machine perfusion has the potential for liver viability testing. (Q18_3)	○	○	○	○
Ex vivo liver machine perfusion provides a platform for potential organ repair strategies. (Q18_4)	○	○	○	○

Q19 The following assess barriers in starting or sustaining an ex vivo machine perfusion program at your institution.

For each one, indicate how much of a barrier it presents where 0 is not a barrier at all and 10 indicates an extreme barrier.

	Not a barrier at all 0 (0)	1 (1)	2 (2)	3 (3)	4 (4)	5 (5)	6 (6)	7 (7)	8 (8)	9 (9)	Extreme barrier 10 (10)
Lack of stakeholder/institutional support (Q19_1)	o	o	o	o	o	o	o	o	o	o	o
Lack of personnel/staff (Q19_2)	o	o	o	o	o	o	o	o	o	o	o
Insufficient expertise (Q19_3)	o	o	o	o	o	o	o	o	o	o	o
Financial burden/cost of the program (Q19_4)	o	o	o	o	o	o	o	o	o	o	o
Procurement staff time burden (Q19_5)	o	o	o	o	o	o	o	o	o	o	o

Page Break

Q20 **Approximately what percentage of your liver transplant patients have Medicare coverage?**

- ○ Less than 20% (1)
- ○ 20 to 50% (2)
- ○ More than 50% (3)
- ○ Unsure (4)

Q21 **Approximately what percentage of your liver transplant patients have private insurance?**

- ○ Less than 20% (1)
- ○ 20 to 50% (2)
- ○ More than 50% (3)
- ○ Unsure (4)

Q22 **If you do not currently have an ex vivo machine perfusion liver transplant program, how likely are you to incorporate this into your practice in the future?**

- ○ Very likely (1)
- ○ Likely (2)
- ○ Unlikely (3)
- ○ Very unlikely (4)
- ○ N/A (5)

Page Break

Q23 **Please share any other thoughts about ex vivo machine perfusion for liver transplants.**

____

____

____

____

____

End of Block: Default Question Block

Start of Block: Submit

Q24 THANK YOU FOR COMPLETING THE SURVEY!

Please click SUBMIT to record your answers.

End of Block: Submit

Table S2: Willingness to Accept or Decline Organ Offers Based on Preservation Method (SCS or NMP) Across Clinical Scenarios with Varying Donor Risk Factors (Age, Graft Type, Distance, and Macrosteatosis) For an Ideal Recipient

***30-year-old DBD donor WITHIN 250 nautical mile acuity circle. Labs and CT imaging are normal. Procurement biopsy with 20 to 30% macrosteatosis.**, n (%)*	
Accept with SCS	69 (85.2%)
Accept with NMP	12 (14.8%)
Decline	0 (0.0%)
***30-year-old DBD donor BEYOND 500 nautical mile acuity circle. Labs and CT imaging are normal. Procurement biopsy with 20 to 30% macrosteatosis.**, n (%)*	
Accept with SCS	40 (49.4%)
Accept with NMP	37 (45.7%)
Decline	4 (4.9%)
***65-year-old DBD donor WITHIN 250 nautical mile acuity circle. Labs and CT imaging are normal. Procurement biopsy with 20 to 30% macrosteatosis.**, n (%)*	
Accept with SCS	36 (44.4%)
Accept with NMP	41 (50.6%)
Decline	4 (4.9%)
***65-year-old DBD donor BEYOND 500 nautical mile acuity circle. Labs and CT imaging are normal. Procurement biopsy with 20 to 30% macrosteatosis.**, n (%)*	
Accept with SCS	14 (17.3%)
Accept with NMP	48 (59.3%)
Decline	19 (23.5%)
***30-year-old DCD donor WITHIN 250 nautical mile acuity circle. Normal labs and imaging are normal. WIT 15 minutes. Procurement biopsy with 20 to 30% macrosteatosis.**, n (%)*	
Accept with SCS	25 (30.9%)
Accept with NMP	50 (61.7%)

Decline	6 (7.4%)
***30-year-old DCD donor BEYOND 500 nautical mile acuity circle. Normal labs and imaging are normal. WIT 15 minutes. Procurement biopsy with 20 to 30% macrosteatosis.**, n (%)*	
Accept with SCS	14 (17.3%)
Accept with NMP	51 (63.0%)
Decline	16 (19.8%)
***55-year-old DCD donor WITHIN 250 nautical mile acuity circle. Normal labs and imaging are normal. WIT 15 minutes. Procurement biopsy with less than macrosteatosis.**, n (%)*	
Accept with SCS	31 (38.8%)
Accept with NMP	44 (55.0%)
Decline	5 (6.3%)
***55-year-old DCD donor WITHIN 250 nautical mile acuity circle. Normal labs and imaging are normal. WIT 15 minutes. Procurement biopsy with 20 to 30% macrosteatosis.**, n (%)*	
Accept with SCS	8 (10.1%)
Accept with NMP	45 (57.0%)
Decline	26 (32.9%)
***55-year-old DCD donor BEYOND 500 nautical mile acuity circle. WIT 15 minutes. Procurement biopsy with less than 5% macrosteatosis.**, n (%)*	
Accept with SCS	19 (23.5%)
Accept with NMP	51 (63.0%)
Decline	11 (13.6%)
***55-year-old DCD donor BEYOND 500 nautical mile acuity circle. WIT 15 minutes. Procurement biopsy with 20 to 30% macrosteatosis.**, n (%)*	
Accept with SCS	4 (4.9%)
Accept with NMP	39 (48.1%)
Decline	38 (46.9%)
***65-year-old DCD donor WITHIN 250 nautical mile acuity circle. WIT 30 minutes. Procurement biopsy with 20 to 30% macrosteatosis.**, n (%)*	
Accept with SCS	3 (3.7%)

Accept with NMP	24 (29.6%)
Decline	54 (66.7%)
***65-year-old DCD donor BEYOND 500 nautical mile acuity circle. WIT 30 minutes. Procurement biopsy with 20 to 30% macrosteatosis.**, n (%)*	
Accept with SCS	0 (0.0%)
Accept with NMP	23 (28.4%)
Decline	58 (71.6%)

Table S3: Differences in Willingness to Accept Organ Offers Based on Preservation Method and Donor Risk Factors. Table S3a shows proportional differences in acceptance decisions (accept on SCS, accept on MP, decline) across donor variables (age, graft type, donor distance, and macrosteatosis). Table S3b shows statistical comparisons of organ offer decisions between preservation strategies and donor risk factors.

A

**Variable**	**Comparison**	**SCS: Difference in Proportion (95% CI)**	**p Value**	**MP: Difference in Proportion (95% CI)**	**p Value**	**Decline: Difference in Proportion (95% CI)**	**p Value**
Age (years)	55 or 65 vs. 30	-0.284 (-0.341, -0.227)	<0.001	0.115 (0.050, 0.181)	<0.001	0.169 (0.119, 0.219)	<0.001
Graft type	DCD vs. DBD	-0.432 (-0.512, -0.352)	<0.001	0.321 (0.228, 0.414)	<0.001	0.111 (0.049, 0.173)	<0.001
Donor Distance (miles)	500 vs. 250	-0.108 (-0.154, -0.061)	<0.001	0.027 (-0.029, 0.082)	0.350	0.081 (0.042, 0.120)	<0.001
Macrosteatosis (%)	20-30% vs. <5%	-0.235 (-0.310, -0.160)	<0.001	-0.067 (-0.179, 0.045)	0.240	0.303 (0.214, 0.392)	<0.001

B

**Variable**	**Comparison**	**SCS vs. NMP**	**SCS vs. Declined**	**NMP vs. Declined**
Age	55/65 vs. 30	<0.001	<0.001	0.100
DCD/DBD	DCD vs. DBD	<0.001	<0.001	0.004
Distance	500 vs. 250	0.010	<0.001	0.409

Macrosteatosis	20-30% vs. <5%	0.046	<0.001	0.015
